# Trends in older adults boarding in US EDs

**DOI:** 10.1093/geroni/igaf122.3349

**Published:** 2025-12-31

**Authors:** Natalia Sifnugel, Daniel Cruz, Molly Jeffery, Rohit Sangal, Brendan Carr, Scott Dresden, Cameron Gettel, Ula Hwang

**Affiliations:** NYU Langone Medical Center, New York, New York, United States; Northwestern Memorial Hospital, Chicago, Illinois, United States; Mayo Clinic, Rochester, Minnesota, United States; Yale University, New Haven, Connecticut, United States; Mayo Clinic Rochester, Rochester, Minnesota, United States; Northwestern University, Evanston, Illinois, United States; Yale School of Medicine, New Haven, Connecticut, United States; NYU Grossman School of Medicine, New York, New York, United States

## Abstract

Inpatient boarding (holding patients in the emergency department [ED] while awaiting an inpatient bed) is associated with increased risk of delirium, medication safety events, and hospital mortality in older adults. We investigated boarding trends across age strata to determine if older patients had elevated rates compared to younger patients. Administrative data for ED encounters resulting in admission from 3 healthcare systems (2 Midwest, 1 Northeast) from 1/1/19 to 12/31/24 were reviewed. Age was categorized by strata: < 21, 21-64, 65-74, 75-84, and ≥ 85. Based on the CMS Age Friendly Hospital Measures, the proportion of patients boarding for 3+ hours was calculated within each year and age strata. Boarding rates varied substantially by system and age group. At Systems 1 (S1) and 3 (S3), ≥ 85 patients had the highest rates (S1: 54.9%, S3: 70.9% in 2024) and, at System 2 (S2), all adult patients (21+) had similar rates (20.5-22.4% in 2024). Boarding rates increased in all systems over the study period. At S1 and S3, < 21 patients had the highest average yearly increase in boarding (S1: 7.4%, S3: 3.9%). At S2, boarding increased most in ≥ 85 (23.9%). In this multisystem evaluation, approximately one in two older adults experience ED boarding, with the oldest patients (≥ 85) facing the greatest exposure to boarding in recent years. These findings taken alongside known delays and safety risks associated with boarding and patient care complexity amongst the oldest adults may result in disproportionate harm to some of the most vulnerable patients.

